# Supplementary material for: Evaluation of the mental health impacts of Universal Credit: protocol for a mixed methods study
Source: BMJ Open. 2022 Apr 8;12(4):e061340. doi: 10.1136/bmjopen-2022-061340 (PMC8996017; doi:10.1136/bmjopen-2022-061340)
Supplement: Author's manuscript [file bmjopen-2022-061340.draft_revisions.pdf]

BMJ Open

BMJ Open is committed to open peer review. As part of this commitment we make the peer review history of every article we publish publicly available.

When an article is published we post the peer reviewers' comments and the authors' responses online. We also post the versions of the paper that were used during peer review. These are the versions that the peer review comments apply to.

The versions of the paper that follow are the versions that were submitted during the peer review process. They are not the versions of record or the final published versions. They should not be cited or distributed as the published version of this manuscript.

BMJ Open is an open access journal and the full, final, typeset and author-corrected version of record of the manuscript is available on our site with no access controls, subscription charges or pay-per-view fees (http://bmjopen.bmj.com).

If you have any questions on BMJ Open's open peer review process please email info.bmjopen@bmj.com

BMJ Open

EVALUATION OF THE MENTAL HEALTH IMPACTS OF UNIVERSAL CREDIT: PROTOCOL FOR A MIXED METHODS STUDY

Journal:	*BMJ Open*
Manuscript ID	bmjopen-2022-061340
Article Type:	Protocol
Date Submitted by the Author:	24-Jan-2022
Complete List of Authors:	Craig , Peter ; University of Glasgow, MRC/CO Social and Public Health Sciences Unit<br>Barr, Benjamin; University of Liverpool, Department of Public Health, Policy and Systems<br>Baxter, Andy; University of Glasgow, MRC/CO Social and Public Health Sciences Unit<br>Brown, Heather; THL; Newcastle University, Population Health Sciences Institute<br>Cheetham, Mandy; Northumbria University, Nursing, Midwifery and Health; North East and North Cumbria Applied Research Collaboration,<br>Gibson, Marcia; University of Glasgow, MRC/CO Social and Public Health Sciences Unit<br>Katikireddi, Srinivasa; University of Glasgow, MRC/CSO Social & Public Health Sciences Unit<br>Moffatt, Suzanne; Newcastle University, Institute of Health & Society<br>Morris, Steph; Newcastle University<br>Munford, Luke; University of Manchester, Manchester Centre for Health Economics<br>Richiardi, Matteo; University of Essex, Centre for Microsimulation and Policy Analysis<br>Sutton, Matt; University of Manchester,<br>Taylor-Robinson, David; University of Liverpool,<br>Wickham, Sophie; University of Liverpool, Department of Public Health, Policy and Systems<br>Xiang, Huasheng; Newcastle University, Population Health Sciences Institute<br>Bambra, Clare; Newcastle University
Keywords:	PUBLIC HEALTH, HEALTH ECONOMICS, QUALITATIVE RESEARCH, MENTAL HEALTH

SCHOLARONE™
Manuscripts

EVALUATION OF THE MENTAL HEALTH IMPACTS OF UNIVERSAL CREDIT: PROTOCOL FOR A MIXED METHODS STUDY

Peter Craig, MRC/CSO Social and Public Health Sciences Unit, University of Glasgow, Berkeley Square, 99 Berkeley Street, Glasgow G3 7HR, UK, peter.craig@glasgow.ac.uk*

Ben Barr, Department of Public Health, Policy and Systems, University of Liverpool, UK

Andrew Baxter, MRC/CSO Social and Public Health Sciences Unit, University of Glasgow, UK

Heather Brown, Finnish Institute of Health and Welfare, Finland

Mandy Cheetham, Department of Nursing, Midwifery and Health, Northumbria University, UK

Marcia Gibson, MRC/CSO Social and Public Health Sciences Unit, University of Glasgow, UK

Srinivasa Vittal Katikireddi, MRC/CSO Social and Public Health Sciences Unit, University of Glasgow,UK

Suzanne Moffatt, Population Health Sciences Institute, Newcastle University, UK

Stephanie Morris, Population Health Sciences Institute, Newcastle University, UK

Luke Munford, Health Organisation, Policy and Economics Research Team, University of Manchester, UK

Matteo Richiardi, Centre for Microsimulation and Policy Analysis, University of Essex, UK

Matt Sutton, Health Organisation, Policy and Economics Research Team University of Manchester, UK

David Taylor Robinson, Department of Public Health, Policy and Systems, University of Liverpool, UK

Sophie Wickham, Department of Public Health, Policy and Systems, University of Liverpool, UK

Huasheng Xiang, Population Health Sciences Institute, Newcastle University, UK

Clare Bambra, Population Health Sciences Institute, Newcastle University, UK

*Corresponding author

Word count: 4490

For peer review only

ABSTRACT

Introduction

The UK social security system is being transformed by the implementation of Universal Credit, which combines six existing benefits and tax credits into a single payment for low-income households. Despite extensive reports of hardship associated with the introduction of Universal Credit, no previous studies have comprehensively evaluated its impact on mental health. Because payments are targeted at low-income households, impacts on mental health will have important consequences for health inequalities.

Methods and analysis

We will conduct a mixed methods study. Work package 1 will compare health outcomes for new recipients of UC with outcomes for legacy benefit recipients in two large population surveys, using the phased rollout of UC as a natural experiment. We will also analyse the relationship between the proportion of UC claimants in small areas and a composite measure of mental health. WP2 will use data collected by Citizen's Advice to explore the socio-demographic and health characteristics of people who seek advice when claiming UC and identify features of the claim process that prompt advice-seeking. WP3 will conduct longitudinal in-depth interviews with UC claimants in England and Scotland to explore reasons for claiming and experiences of the claim process. Staff supporting claimants will also be interviewed. WP4 will use a dynamic microsimulation model to simulate the longer-term health impacts of different implementation scenarios. WP5 will undertake cost-consequence analysis of the potential costs and outcomes of introducing UC and cost-benefit analyses of mitigating actions.

Ethics and dissemination

We obtained ethical approval for the primary data gathering from the University of Glasgow, College of Social Sciences Research Ethics Committee, application number 400200244.

Registration details

The study is registered with the Research Registry (https://www.researchregistry.com/), with the reference: researchregistry6697.

Keywords

Universal Credit, mental health, mixed methods

For peer review only

STRENGTHS AND LIMITATIONS OF THIS STUDY

- The study makes use of a natural experiment generated by the phased implementation of a major UK welfare reform, the introduction of Universal Credit, which allows us to compare UC recipients with recipients of legacy benefits prior to UC implementation.
- We will use unique data from our partnership with Citizen's Advice alongside longitudinal qualitative interviews, photo elicitation methods and in-depth interviews with staff involved in supporting them, to explore the experiences of UC recipients and to inform the detailed design of the quantitative analyses and modelling work.
- We will incorporate effect estimates into a dynamic microsimulation model to explore the health impacts of alternative ways of delivering UC and how such impacts vary across population subgroups.
- We will conduct cost-consequence analyses to quantify the costs and outcomes of implementing UC and cost-benefit analyses of measures to mitigate any observed harms.
- We consulted extensively with UC recipients and organisations representing and providing support for UC recipients in designing the study to ensure that we address questions that matter to UC recipients and organisations that support them. Public involvement and engagement will be an ongoing element of the study.

For peer review only

INTRODUCTION

Social security policies account for up to one quarter of GDP and 40% of public expenditure in high income countries[1] and are a key determinant of income and health inequalities.[2] In the UK, Universal Credit (UC) is transforming social security for working age people by combining six existing benefits and tax credits (known as legacy benefits) into a single monthly payment. UC is designed to improve work incentives for people on low incomes but has been criticised for causing hardship due to the job-seeking requirements and other conditions related to eligibility, and to the way that claims are managed and payments made. UC is being introduced gradually, with rollout due to be complete in 2024.[3] Research to date has not provided a comprehensive picture of UC's impact on population health or health inequalities. Our proposed study will be the first to do so, by examining the impact of the introduction of UC on mental health and wellbeing, and how these effects are moderated by variation in implementation across England, Wales and Scotland.

In preparing our proposal we updated and extended the literature searches from a recently published study of the impact of UC on psychological distress[4] by members of our team, to include papers published in 2019-20. We searched six bibliographic databases (MEDLINE (number of hits=0), PubMed (5), Scopus (5), PsycInfo (0), Social Science Citation Index (8), EconLit (3), using the terms ("universal credit" AND "mental health" OR "wellbeing" OR "well-being" OR depress* OR anxiety OR "psychiatric disorder*" OR "common mental disorder*" OR "psych* morbidity"). We also searched two preprint servers (Socarxiv (2), Medarxiv (1). We found 17 papers, but no new studies, other than our own,[2] that dealt with the health impacts of UC.

Several reports from public bodies draw attention to possible health harms. Based on evidence from local authorities and charities, the UK Parliament's Public Accounts Committee has raised concerns about financial hardship associated with the five week wait for the first payment, compounded by delays in processing new claims.[5] The UN Special Rapporteur on extreme poverty and human rights highlighted difficulties faced by 'poorer and more vulnerable households' in negotiating the online application process, concluding that 'UC was harming many claimants' mental health, finances, and work prospects.'[6] Doctors' organisations have reported increased consultations in General Practices following the transition to UC.[7] Significant concerns have been raised about the impact of UC on rising child poverty in the UK.[8] Qualitative studies have reported 'widespread and deeply negative impacts on wellbeing.'[9] In our own research,[10, 11] claimants reported that the threat of sanctions, delays in payment and financial insecurity associated with UC harmed their physical and mental health. There has been one quantitative study of health impacts to date, again by members of our team.[4] This study compared changes in mental health among unemployed

For peer review only - http://bmjopen.bmj.com/site/about/guidelines.xhtml

people in areas where UC had been implemented with areas where legacy benefits remained in place, finding a seven-percentage point increase in psychological distress in the former.

Although existing evidence suggests mainly adverse effects, UC may improve health if it provides a higher or more dependable income for recipients than existing benefits, or if it supports paid work. Research conducted by the Department for Work and Pensions' (DWP) found that more intensive support for job-seeking under UC increased earnings[12] and helped unemployed claimants return to work sooner.[13]

To date, the evidence of health impacts relates to a period when UC had only been implemented for some groups of new claimants. The overall health and equity impacts may change markedly as legacy benefit recipients migrate to UC. Substantial but temporary changes in the way UC is administered were made in response to the economic shock associated with the COVID-19 lockdown measures in early 2020. Conditionality and some forms of debt recovery were suspended for three months and have since remained well below pre-pandemic levels. The standard cash allowance (the most a household could receive, before taking income and savings into account) was raised by £1000 per annum from April 2020 until October 2021[14] A very large number of new claims were made and processed in the early months of the lockdown. Claims rose 9-fold, to 500,000 per week in early April 2020, and the caseload doubled from 3 to 6 million, before new claims returned to previous levels by July 2020.[15] A new cohort of UC recipients has been created whose composition is likely to differ significantly from the pre COVID-19 caseload (for example, they are less likely to have received benefits in the past and more likely to have savings).[16] As well as estimating the impact of UC on population mental health, we shall explore the experiences of these new recipients to gain insights into whether differences in health impacts might be expected.

Our objectives are: (1) to measure the impacts on mental health and wellbeing among adults and children affected by UC, compared to a legacy-benefits comparator group; to explore how impacts are distributed and effects vary between England/Wales and Scotland, and the implications of the COVID-19 related changes to UC for the health of recipients; (2) to identify features of the UC claim and payments process associated with health difficulties for claimants, and to explore inequalities within and between regions in the pattern of advice-seeking from Citizens Advice; (3) to explore the ways in which the experience of claiming and managing UC affects claimants' health, and how experiences differ between England/Wales and Scotland; (4) to develop a microsimulation model to investigate possible income, employment and health consequences of differing UC implementation scenarios, up to ten years post-implementation; (5) to measure the cost-consequence of UC, analyse

For peer review only

its distributional consequences and conduct cost-benefit analyses of moderating/mitigating measures implemented in Scotland or by specific Local Authorities.

METHODS AND ANALYSIS

Study design

We will undertake a mixed methods study with five closely linked work packages. Work package (WP) 1 will take a natural experimental approach to identify the effects of UC on mental health and wellbeing using two large-scale population surveys, the UK Household Longitudinal Study (UKHLS)[17] and the Annual Population Survey (APS).[18] We shall use the information about the phased implementation of UC that is made publicly available via the DWP's Stat-Xplore system[19] to define exposed and unexposed groups and compare changes in the health of survey respondents exposed to UC, with changes in the health of respondents living in areas where legacy benefits still apply. We will also use a longitudinal neighbourhood dataset for 42,000 small areas (Lower Layer Super Output areas in England and Wales; Datazones in Scotland), the NIHR North West Coast Applied Research Collaboration – Place-based Longitudinal Data Resource.[20] The dataset includes monthly measures indicating which population groups (e.g., new claimants, existing claimants, people with/ without children, people with disabilities), were affected by the phased rollout of UC in each neighbourhood in each time period. To identify any area-level mental health effects of UC, we will utilise a composite neighbourhood mental health measure – the Small Area Mental Health Index (SAMHI), available annually for the same small areas, but adapted to include Scotland.[21] Using the COVID-19 waves of the UKHLS, we shall compare mental health among new UC claimants, other respondents who have experienced income losses and existing UC claimants. These analyses will be primarily descriptive rather than designed to formally identify impacts.

WP2 will analyse administrative data collected by Citizens Advice (CA) on the characteristics of people seeking help in connection with UC claims between 2017-2020, a survey conducted in the North East of England on financial stress associated with the UC application process in September/October 2019, and a linked dataset derived from the Liverpool 'advice on prescription' service, which combines CA data on advice-seeking with information about use of health and social care services.

WP3 comprises two qualitative studies: (i) a longitudinal qualitative and photovoice[22] study of UC claimants and (ii) qualitative interviews with DWP JobCentre Plus staff who support claimants through the process of claiming and front line staff from education, local government, housing and the voluntary and community sectors who support people claiming UC. We will seek to interview

For peer review only

claimants at two timepoints, 18-24 months apart, to explore how the experience of claiming and receiving UC is influenced by other changes in life circumstances, such as transitions into or out of work, changes in relationship status, birth of children or changes in housing arrangements. Use of photovoice methods, alongside more conventional qualitative methods, will enable the follow-up interviews to be participant-led.

WP4 will develop a stochastic dynamic microsimulation model of the longer-term health impacts of UC, building on an existing static microsimulation model of income and welfare policy (UKMOD, www.microsimulation.ac.uk/ukmod), and incorporating information about effects and mediators from the other project work packages. We shall use the model to simulate the possible health impacts of different UC implementation scenarios, and to explore how these differ between subgroups of the working age population, defined by personal characteristics, household type or geography.

WP5 will undertake a cost-consequence analysis of Universal Credit (UC) and assess its distributional impacts by health, income, and other individual and area-level characteristics. We will identify and estimate the magnitude of the wide range of potential positive and negative consequences of introducing UC. We will provide, where appropriate, monetarised valuations of these effects and detail which sectors and government departments experience them. Costs and consequences associated with implementing UC will be compared to a baseline scenario of continued legacy benefits. We will also undertake cost-benefit analyses of a range of actions that have been implemented in Scotland or locally within England to mitigate possible negative impacts of implementing UC.

Intervention

UC combines six existing benefits and tax credits for people who are unemployed or on a low income into a single monthly payment. It is intended to encourage an early return to work by incorporating support for job-seeking and removing disincentives associated with high marginal withdrawal rates under the legacy benefits. The differences between UC and the legacy benefits are summarised in figure 1.

Figure 1 here

[UC began to be used for new claims in October 2013, with rollout for new unemployed claimants complete by December 2018. Existing claims for legacy benefits and tax credits will be transferred to UC in a process of 'managed migration'. The process began with a geographically limited pilot which

For peer review only

was suspended early in 2020, but is expected to recommence in 2022, although how migration will now be managed is not yet known.

UC may improve recipients' health via increased income and employment, but some features of its design and delivery may be harmful. The application of conditionality and sanctions, the minimum five week wait for the first payment, which may in practice be much longer,[3] the two child limit and benefit cap, and the inclusion of support for housing costs within a single monthly household-level payment made to one individual, have all attracted concern. Some households, such as working families living in rented accommodation, will have higher incomes from UC than they would receive from legacy benefits, but others, including people receiving disability benefits, self-employed people with low earnings, and some working lone parents who were formerly receiving tax credits, may lose income.[23] UC is a UK-wide system but in Scotland, under arrangements known as 'Scottish Choices,' claimants can opt for two-weekly payments, or to have the housing element paid direct to their landlord.[24, 25]

The effects on health of the transition to UC are therefore likely be complex and geographically variable, with some households benefitting from improved living standards and greater financial security, but others facing an increase in hardship, insecurity and debt.[26] Effects may be direct, for example by increasing stress, or be mediated by changes in behaviours such as smoking, drinking, or criminal activity.[27] Children's health likewise will be affected directly by changes in household living standards,[28] for example in relation to diet or warmth, but also by effects operating through parental stress and consequent changes in parenting and other behaviours.[29]

As noted above, substantial but temporary changes in the way UC is administered have been made in response to the economic shock associated with the COVID-19 lockdown measures. As well as estimating the overall impact on UC on population mental health, we shall explore the experiences of these new UC recipients to gain insights into whether differences in health impacts might be expected.

Sample selection

In WP1 we will analyse data on people of working age and their children from 2009-2022 in the UKHLS, and on people of working age from 2011-2024 in the APS. The UKHLS and APS use stratified clustered random probability samples of the UK population. The CA surveys (WP2) include adults in England who have contacted CA for assistance with claiming UC from 2017 onwards. For a sub-sample of respondents from North East England a survey was conducted by CA was in August 2019 and a follow up questionnaire was administered in October 2019 to all individuals who had made a UC claim to understand the characteristics of people who experience financial hardship. The 'advice

For peer review only - http://bmjopen.bmj.com/site/about/guidelines.xhtml

on prescription service' data is available for people engaging with CA Liverpool from 2014, but linked data will span 2010 to 2023.

The samples of UC claimants for WP3 will be drawn from two large urban areas, Tyne and Wear (population 1.1 million) in North East England and the Glasgow City Region (population 1.2 million) in Scotland. A number of recruitment strategies will be used, including via gatekeepers[8, 9] in public, voluntary and community sector organisations, workplaces and trades unions, and via social media and leaflets placed in community locations such as libraries. We will aim for a maximum variation sample, taking account of age, gender, ethnicity, household composition, education level, health, disability, employment and housing status. The sample will include people who have been on UC for some time, new claimants and those transferring to UC from legacy benefits. We will include people who were claiming UC prior to March 2020, people who claimed UC during the UK's lockdown period and those who have become claimants later due to the economic impact of the pandemic. We will recruit staff from a range of organisations in each locality via our extensive professional networks in each conurbation.

Sample size and statistical power

The UKHLS includes approximately 40,000 households who are surveyed annually. The 2009-2022 survey datasets will include approximately 280,000 observations on around 52,000 adults and 30,000 observations on 5,000 children. To identify the impact of UC, we will conduct difference-in-difference (DiD) analyses, comparing members of households newly exposed to UC as rollout proceeds, with households who are unlikely to be exposed. Focusing on child health outcomes, because the numbers available are smaller, in the UKHLS datasets from 2009-18 we will have 300 children in the intervention group and 3300 in the control group, providing 80% power to detect an 8% relative increase in SDQ scores. This is a markedly smaller effect than the 20% relative increase in the prevalence of psychological distress we found in our previous study[4] of the impact of Universal Credit on adult mental health up until 2018, so provides a conservative estimate of the power of the proposed study. The sample size in the UKHLS COVID-19 surveys is approximately 10,000, of whom approximately 4,000 reported substantial (>5%) income losses, and around 300 reported new UC claims in the April survey wave. Currently, nine waves of data are available, from April 2020 to September 2021, with questions about UC included in waves 1, 2, 4, 6, 8 and 9.[30]

The APS collects self-assessed health and wellbeing data on approximately 180,000 working age people per year, so provides good power for England-Scotland comparisons and other subgroup analyses. Our analysis of area level mental health effects will utilise annual data from a longitudinal

For peer review only

dataset of 42,000 neighbourhoods from 2011 to 2022, or approximately 504,000 neighbourhood-years.

The CA survey dataset contains information on approximately 280,000 people who seek advice. The longitudinal survey contains information on 250 people, with a response rate of 85%. The advice on prescription dataset contains information on approximately 5000 people each year since 2014.

For the qualitative study, we will recruit up to 80 UC claimants aged 18–66 (state pension age), divided equally between the two study sites. We will seek to re-interview 40 first round respondents 18-24 months after their first interview, a sub-sample of interviewees using photovoice methods.[22] We will aim to interview up to 15 staff from a range of organisations in each study site.

WP4 and 5 draw on data from WP1-3 above and from the additional sources listed in the analysis plan below.

Outcomes

Our primary focus is on mental health and wellbeing. Mental health is an important dimension of overall health and wellbeing, that is sensitive to the effects of changes in employment or income,[31, 32] and an important determinant of physical health. Parental mental health is also an important mediator of the impact of poverty on child health.[33] In the survey analyses (WP1) we shall use standard measures of mental health and wellbeing, such as the Mental Component Summary (MCS) of the 12-item Short-Form Health Survey (SF-12) for adults, the Strengths and Difficulties Questionnaire (SDQ) for children,[34] and the Office for National Statistics (ONS) ONS4 measure of wellbeing (life satisfaction, sense that life is worthwhile, happiness and anxiety).[35] Secondary outcomes include other mental and physical health measures available in the surveys. In the neighbourhood-level analyses we will use a composite mental health measure, the Small Area Mental Health Index (SAMHI).[21] In the CA surveys in WP2 we shall use presence of a self-reported long-term mental health condition. WP4 and WP5 will use similar outcomes to WP1, and WP5 will also explore the costs and non-health consequences of implementing UC. In WP3 we shall explore other health outcomes identified as important by respondents.

Analysis plan

To identify the effects of UC in the UKHLS and APS datasets (WP1) we will fit DiD models, with terms for group and period to define respondents who are exposed or unexposed to UC, depending on their socio-demographic characteristics, location and date of interview, and a group by period interaction term to identify the effect of UC. We will add individual and area-level covariates to account for time-varying confounders[36] and fit mixed-models to account for clustering of

For peer review only

observations within households and areas. Sensitivity analyses will be conducted using alternative definitions of exposed and unexposed populations. We will test the validity of the key identifying assumptions, such as common pre-intervention trends, and use additional (e.g., matching) methods if needed. We will also conduct falsification tests using placebo outcomes to test for specificity of effects.

For the SAMHI analyses we will use fixed-effect panel regression methods to investigate whether increases in UC in each LSOA are associated with changes in mental health problems, while controlling for time-varying confounders (e.g. local government expenditure)[37].We will check the robustness of the results by using alternative ways of estimating effects, such as continuous DiD and synthetic control methods[38] to compare outcomes in areas where UC has been introduced and areas not yet exposed.

To investigate the extent to which changes in income, employment status, and financial housing difficulties (such as rent arears) mediate the effects of the introduction of UC on health outcomes, we will undertake causal mediation analysis adjusting for time-varying confounding.[39, 40, 41] Subgroup analysis will investigate differences in health impacts between Scotland and the rest of GB, or between areas with differing labour market characteristics. By stratifying the analysis before, during and after the pandemic we will investigate whether moving onto UC had the same effect during these different time periods, when the policy and wider social context were very different. Using the COVID-19 waves of the UKHLS, we shall compare mental wellbeing among new UC claimants, other respondents who have experienced income losses and existing UC claimants. These analyses will be primarily descriptive rather than designed to formally identify impacts.

In WP2 we will carry out descriptive analyses of the England-wide CA survey data to summarise the health and socio-demographic characteristics of people seeking advice in applying for UC, and explore differences at the local authority level in key demographic and health characteristics of advice seekers. We will use the longitudinal CA dataset to describe the socio-demographic and health characteristics of people who are most and least likely to experience financial insecurity as a result of the five week wait for the first payment. We will also apply longitudinal multivariable regression to the 'advice on prescription' linked dataset, similar to the methods utilised in WP1. We will use the cross-sectional national data from March 2020 onwards to explore changes in the socio-demographic and health profile of people seeking advice with UC claims following the COVID-19 pandemic.

In WP3, we will apply thematic content analysis to the longitudinal qualitative data,[42] using NVivo software to assist data management, retrieval and sharing between members of the research team.

For peer review only

A comparative analysis across time, locality and participant characteristics will be undertaken to identify possible pathways linking the experience of claiming UC with health outcomes, and how they vary between claimants and between study sites. This will enable identification of differences of experience by locality and in-depth analysis of characteristics including gender, age, and employment status, as well as facilitating comparisons of the experiences of longer term (pre-COVID) and post-COVID claimants. Photovoice data will be coded and incorporated within the analysis of the interview material.[22] Interviews with staff will also be analysed thematically.

In WP4 we will first create a dynamic, synthetic population that reflects the GB population, using data from UKHLS, APS, census data, population projections, mortality estimates and the Family Resources Survey. We will develop a new stochastic dynamic microsimulation model based on the existing UKMOD income and welfare policy model,[43] coupled with estimates of the relationship between UC, income, employment and health. We will use the model to simulate the effects on health of different UC implementation scenarios compared with three baseline scenarios: the continuation of legacy benefits; UC as it existed in the early months of the COVID-19 pandemic; and UC in its contemporary form (i.e., at the time of running the model). Implementation scenarios will include varying levels of standard allowance, conditionality, payment frequency, etc. Under each scenario, we will transition the synthetic population forward in one-year increments for up to ten years, so that we can compare the effects on health and health inequalities of the different policy choices. We will check the robustness of the model by comparing simulated health outcomes with known outcomes from UKHLS waves that were not used to parameterise the model and from other surveys such as the Scottish Health Survey. To allow for the uncertainty in the sample-based parameter estimates used to build the model, we will use Monte Carlo simulation (10,000 runs) to derive 95% credible intervals.

WP5 comprises three linked analyses: ex post cost-consequence and distributional cost-consequence analyses of the implementation of UC, and an ex ante cost benefit analysis of measures to mitigate against possible adverse effects of UC. We will follow HM Treasury Green Book[44] guidelines for economic evaluation, to enable comparability with other evaluations of UK Government policies. We will obtain measures of costs of implementing legacy benefits, UC and mitigating measures through liaison with project partners. We will consider a wide range of consequences, informed by liaison with stakeholders and the findings of WPs 1-3. They will include, but not be limited to, health and wellbeing; income (at both individual and household level); employment; economic productivity (Gross Value Added); and use of health and care services. We will use estimates of the short term effects of UC from WP1, supplemented by additional analyses using similar methods as far as possible, but considering alternatives (e.g. lagged dependent

For peer review only

variables, synthetic controls) where DiD assumptions are not met.[45, 46] Economic, health and wellbeing outcomes will be monetarised where appropriate using standard methods[47, 48] The initial time horizon for the accrual of costs and consequences will be ten years (consistent with WP4), though we will consider alternative time-horizons informed by discussions with stakeholders.

Public Involvement and Engagement

In designing the study we consulted widely with organisations representing and providing support for UC claimants. Strong public involvement and engagement (PIE) will be maintained throughout the study using a range of methods and with specialist support from the North East and North Cumbria Applied Research Collaboration. We define 'public' to include current or former UC claimants, users and staff of advice and support services that help UC claimants, and policy makers and practitioners with working knowledge of UC implementation. We shall seek their advice on changes in UC policy and delivery, aspects of research methods, including data gathering, analysis, interpretation and reporting, and their active engagement in the development of payment policies and procedures, fieldwork documents, analysis plans and wider dissemination. Engagement will be co-ordinated by a named PIE lead (MC), and the whole study team will work according to an agreed PIE values framework.

ETHICS AND DISSEMINATION

Ethics

The University of Glasgow is the study sponsor. We have obtained ethical approval for the primary data gathering in WP3 from The University of Glasgow College of Social Sciences Research Ethics Committee, application number 400200244. We are using a Special Licence version of the APS datasets, under the UK Data Service Secure Lab Project No. 116676.

Dissemination

As well as presenting findings at academic meetings and publishing findings in peer-reviewed journals we will use our networks to actively disseminate findings to UC claimants, the wider public, practitioners and policy-makers. Specific impact-focused activities will include: (1) Co-production of non-academic outputs for national and local government actors, provider organisations and advocacy groups e.g. text and video narrative summaries; 'policy briefings'; infographics; research digests; (2) Photo/text exhibition for a national public engagement tour of libraries, community centres, museums, galleries; (3) Targeted, interactive work-in-progress and policy briefing sessions for varied policy and practice audiences; (4) Stakeholder workshops and learning events for key

For peer review only

interest groups; (4) Joint research meetings involving contributions from academics, policy and practice and public; (5) A purpose-built website to disseminate results via policy briefs, blogs and infographics (6) 'open source' versions of the WP4 policy models to enable government policy analysts to use them to inform future decision-making and for other researchers to apply our methodology into other areas; (7) Public engagement sessions via science festivals and various university initiatives e.g. Pint of Science, Café Scientifique; (8) Social media and traditional media press releases of key results.

Contributorship statement

PC wrote the first draft of the protocol. All authors contributed to revising the manuscript and approving the final version for publication.

Competing interests

The authors declare no competing interests.

Funding

This research is supported by the UK National Institute of Health Research Public Health Research Programme, grant number NIHR131709. PC, MG and SVK acknowledge funding from the UK Medical Research Council (MC_UU_00022/2) and Scottish Government Chief Scientist Office (SPHSU17). SVK acknowledges funding from a NRS Senior Clinical Fellowship (SCAF/15/02) and the European Research Council (949582). CB and MC acknowledge funding from ARC North East North Cumbria (NIHR200173), and CB from the National Prevention Research Initiative for the Systems science in Public Health and Health Economics Research consortium (MR/S037578/1). LM and MS acknowledge funding from ARC Greater Manchester (NIHR200174). SW acknowledges funding from a Wellcome Trust Society and Ethics Research Fellowship (200335/Z/15/Z). BB and DT-R are funded by the National Institute for Health Research (NIHR) School for Public Health Research (SPHR), Grant Reference Number PD-SPH-2015. DT-R is also funded by the Medical Research Council (MRC) on a Clinician Scientist Fellowship (MR/P008577/1). BB is also supported by the National Institute for Health Research Applied Research Collaboration North West Coast (ARC NWC).

For peer review only

REFERENCES

[1] Eurostat. Government expenditure on social protection. https://ec.europa.eu/eurostat/statistics-explained/index.php?title=Government_expenditure_on_social_protection (last accessed 6 January 2022).

[2] Mackenbach JP, Bopp M, Deboosere P, et al. Determinants of the magnitude of socioeconomic inequalities in mortality: A study of 17 European countries. Health Place 2017;4744-53.

[3] National Audit Office. Rolling out Universal Credit. 2018. https://www.nao.org.uk/wp-content/uploads/2018/06/Rolling-out-Universal-Credit.pdf (last accessed 6 January 2022).

[4] Wickham S, Bentley L, Rose T, et al. Effects on mental health of a UK welfare reform, Universal Credit: a longitudinal controlled study. Lancet Public Health, 2020;5:e157-e164.

[5] House of Commons Committee of Public Accounts. Universal Credit. Sixty-Fourth Report of Session 2017-19. HC 1183, 26 October 2018.

[6] Statement on Visit to the United Kingdom, by Professor Philip Alston, United Nations Special Rapporteur on extreme poverty and human rights. London, 16 November 2018.

[7] Arie S, Doctors' concerns over universal credit are mounting. BMJ 2018;363.

[8] Child Poverty Action Group. Universal Credit: What needs to change to reduce child poverty and make it fit for families? CPAG, June 2019. https://cpag.org.uk/sites/default/files/files/policypost/Universal%20credit%20-%20what%20needs%20to%20change_0.pdf (last accessed 20/01/2022).

[9] Wright S, Dwyer P, Jones K, et al. Final findings: universal credit. Welfare Conditionality, Sanctions, Support and Behaviour Change. 2018. http://www.welfareconditionality.ac.uk/wp-content/uploads/2018/05/40414-Universal-Credit-web.pdf

[10] Cheetham M, Moffatt S, Addison M, et al Impact of Universal Credit in North East England: a qualitative study of claimants and support staff. BMJ Open 2019;9:e029611.

[11] Cheetham M, Gorman S, Gibson E, et al. "It's not about telling people to eat better, stop smoking or get on the treadmill." In: Newbury-Birch D and Allan K, eds. *Co-creating and co-producing Research Evidence. A Guide for practitioners and academics in health, social care and education settings.* Abingdon: Routledge, 2020.

[12] Department for Work and Pensions. Universal Credit: in work progression randomised controlled trial. https://www.gov.uk/government/publications/universal-credit-in-work-progression-randomised-controlled-trial (last accessed 20/01/2022).

[13] Department for Work and Pensions. Estimating the Early Labour Market Impacts of Universal Credit. DWP Ad Hoc Research Report No. 19. February 2015.

[14] House of Commons Library, Coronavirus. Support for Household Finances. 22 June 2020. https://commonslibrary.parliament.uk/research-briefings/cbp-8894/ (last accessed 20/01/2022).[15] Department for Work and Pensions. Universal Credit Statistics: 29 April 2013 to 9 July 2020. https://www.gov.uk/government/statistics/universal-credit-statistics-29-april-2013-to-9-july-2020/ (last accessed 20/01/2022).

[16] House of Lords Economic Affairs Committee. Universal Credit isn't working: proposals for reform 2nd Report of Session 2019-21. HL Paper 105, 31 July 2020 https://publications.parliament.uk/pa/ld5801/ldselect/ldeconaf/105/10502.htm (last accessed 20/01/2022).

[17] Understanding Society. COVID-19. https://www.understandingsociety.ac.uk/topic/covid-19 (last accessed 20/01/2022).

[18] Office for National Statistics. Annual Population Survey (APS) QMI. https://www.ons.gov.uk/employmentandlabourmarket/peopleinwork/employmentandemployeetypes/methodologies/annualpopulationsurveyapsqmi (last accessed 20/01/2022).

[19] Department for Work and Pensions. Stat-Xplore. 2019. https://stat-xplore.dwp.gov.uk/webapi/jsf/login.xhtml (last accessed 20/01/2022).

20 Place-based Longitudinal Data Resource. https://pldr.org/ (last accessed 06/01/2022).

21 Daras K, Barr B (2021), Small Area Mental Health Index (SAMHI) [Open Dataset], Place-based Longitudinal Data Resource, DOI: 10.17638/datacat.liverpool.ac.uk/1188, last accessed 06/01/2022.

[22] Guell C, Ogilvie D. Picturing community: photovoice and seeking well-being in everyday travel. Qual Res 2015;15:201-218.

[23] Brewer M, Joyce R, Waters T et al. Universal credit and its impact on household incomes: the long and the short of it. https://www.ifs.org.uk/publications/14083 (last accessed 20/01/2022).

[24] Universal Credit (Scottish choices). https://www.gov.scot/policies/social-security/universal-credit/ (last accessed 20/01/2022).

[25] Child Poverty Action Group (2019) Universal Credit Scottish Choices. 18/2/19 https://cpag.org.uk/scotland/welfare-rights/scottish-benefits/universal-credit-scottish-choices (last accessed 16.07.20).

[26] Bennett F, Sung S. Money matters: using qualitative research for policy influencing on gender and welfare reform, Innovation: Eur J Soc Sci Res 2014;27:5-19.

[27] d'Este R, Harvey A. Universal Credit and Crime. SSRN, October 14, 2020. https://ssrn.com/abstract=3642502 or http://dx.doi.org/10.2139/ssrn.3642502 (last accessed 20/01/2022).

[28] Stewart K, Reeves A and Patrick R. A time of need: Exploring the changing poverty risk facing larger families in the UK. CASE Working Paper 224, July 2021. https://sticerd.lse.ac.uk/dps/case/cp/casepaper224.pdf (last accessed 20/01/2022).

[29] Pearce A, Dundas R, Whitehead M, et al. Pathways to inequalities in child health. Arch Dis Child 2019;104:998-1003.

[30] Understanding Society. COVID-19. https://www.understandingsociety.ac.uk/documentation/covid-19 (last accessed 10/01/2022)

[31] Reeves A, Fransham M, Stewart K et al.Did the introduction of the benefit cap in Britain harm mental health? A natural experiment approach. CASE Working Paper 221, November 2020. https://sticerd.lse.ac.uk/dps/case/cp/casepaper221.pdf (last accessed 20/01/2022).

[32] Reeves A, McKee M, Mackenbach J et al. Introduction of a national minimum wage reduces depressive symptoms in low wage workers; a quasi-natural experiment in the UK. Health Econ 2016;26:639–655 (last accessed 20/01/2022).

[33] Wickham S, Whitehead M, Barr B, et al. The effect of a transition into poverty on child and maternal mental health: a longitudinal analysis of the UK Millennium Cohort Study. Lancet Public Health 2020; 5: e157–64 [34] Goodman, R. (1997), The Strengths and Difficulties Questionnaire: A Research Note. J Child Psychol Psychiatr 1997;38:581-586.[35] ONS. Personal well-being user guidance. https://www.ons.gov.uk/peoplepopulationandcommunity/wellbeing/methodologies/personalwellbeingsurveyuserguide (last accessed 20/01/2022).

[36] Daniel RM, Cousens SN, De Stavola BL, et al. Methods for dealing with time-dependent confounding. Stat Med 2013;32:1584–618.

[37] Barr B, Taylor-Robinson D, Stuckler D, et al. 'First, do no harm': are disability assessments associated with adverse trends in mental health? A longitudinal ecological study. J Epidemiol Community Health 2016;70:339-345

[38] Robbins MW, Saunders J, Kilmer B. A Framework for Synthetic Control Methods With High-Dimensional, Micro-Level Data: Evaluating a Neighborhood-Specific Crime Intervention. J Am Stat Assoc 2017;112:109–26.

[39] VanderWeele TJ. Explanation in Causal Inference: Methods for Mediation and Interaction. New York: Oxford University Press, 2015.

[40] Imbens GW, Rubin DB. Causal Inference for Statistics, Social and Biomedical Sciences: an Introduction. New York: Cambridge University Press, 2015.

[41] Hernán MA, Robins JM. Estimating causal effects from epidemiological data. Journal of epidemiology and community health 2006;60:578–86.

For peer review only

[42] Neale B What is longitudinal qualitative research ? London: Bloomsbury Academic, 2018

[43] Richiardi M, Collado D and Popova D. UKMOD –A new tax-benefit model for the four nations of the UK. Int J Microsimul, forthcoming. 2021

[44] HM Treasury. The Green book. Central Government Guidance on Appraisal and Evaluation. HM Treasury, 2018. https://assets.publishing.service.gov.uk/government/uploads/system/uploads/attachment_data/file/685903/The_Green_Book.pdf (last accessed 20/01/2022).

[45] O'Neill S, Kreif, N, Grieve, R et al. Estimating causal effects: considering three alternatives to difference-in-differences estimation. Health Serv Outcomes Res Method 2016;16:1–21.

[46] O'Neill S, Kreif N, Sutton M, et al. A comparison of methods for health policy evaluation with controlled pre-post designs. Health Serv Res. 2020;55:328-338.

[47] NICE. How NICE Measures Value for Money in Relation to Public Health Interventions. 2013. https://www.nice.org.uk/Media/Default/guidance/LGB10-Briefing-20150126.pdf (last accessed 20/01/2022).

[48] Shah K, Praet C, Devlin N, et al. Is the Aim of the English Health Care System to Maximize QALYs? J Health Serv Res Policy 2012;17(3): 157–63.

For peer review only

Figure Legends

Figure 1 Universal Credit and legacy benefits (Source: Reference [2])

For peer review only

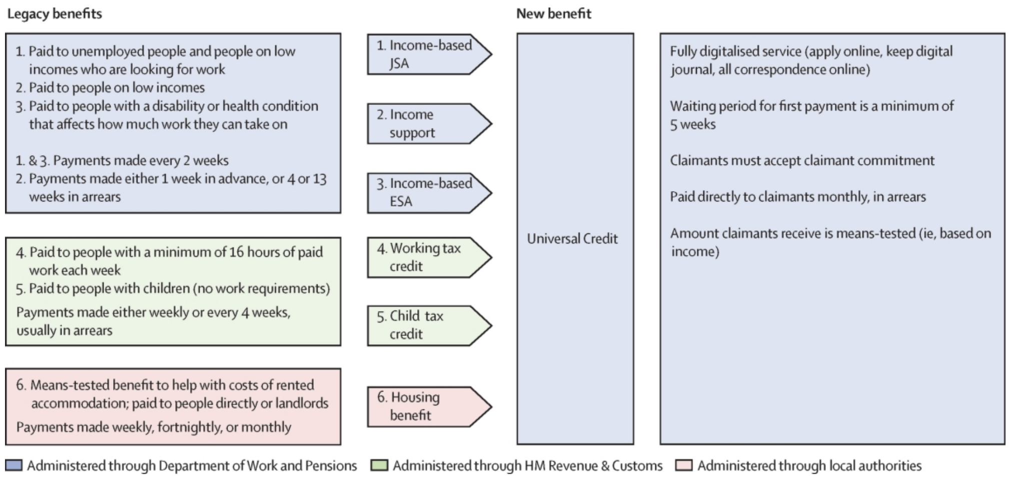

Figure 1 Universal Credit and legacy benefits (Source: Reference [2])

211x98mm (300 x 300 DPI)

BMJ Open

EVALUATION OF THE MENTAL HEALTH IMPACTS OF UNIVERSAL CREDIT: PROTOCOL FOR A MIXED METHODS STUDY

Journal:	*BMJ Open*
Manuscript ID	bmjopen-2022-061340.R1
Article Type:	Protocol
Date Submitted by the Author:	08-Mar-2022

Complete List of Authors:	Craig , Peter ; University of Glasgow, MRC/CO Social and Public Health Sciences Unit<br>Barr, Benjamin; University of Liverpool, Department of Public Health, Policy and Systems<br>Baxter, Andy; University of Glasgow, MRC/CO Social and Public Health Sciences Unit<br>Brown, Heather; THL; Newcastle University, Population Health Sciences Institute<br>Cheetham, Mandy; Northumbria University, Nursing, Midwifery and Health; North East and North Cumbria Applied Research Collaboration,<br>Gibson, Marcia; University of Glasgow, MRC/CO Social and Public Health Sciences Unit<br>Katikireddi, Srinivasa; University of Glasgow, MRC/CSO Social & Public Health Sciences Unit<br>Moffatt, Suzanne; Newcastle University, Institute of Health & Society<br>Morris, Steph; Newcastle University<br>Munford, Luke; University of Manchester, Manchester Centre for Health Economics<br>Richiardi, Matteo; University of Essex, Centre for Microsimulation and Policy Analysis<br>Sutton, Matt; University of Manchester,<br>Taylor-Robinson, David; University of Liverpool,<br>Wickham, Sophie; University of Liverpool, Department of Public Health, Policy and Systems<br>Xiang, Huasheng; Newcastle University, Population Health Sciences Institute<br>Bambra, Clare; Newcastle University

**Primary Subject Heading**:	Public health
Secondary Subject Heading:	Health economics, Mental health
Keywords:	PUBLIC HEALTH, HEALTH ECONOMICS, QUALITATIVE RESEARCH, MENTAL HEALTH

SCHOLARONE™
Manuscripts

EVALUATION OF THE MENTAL HEALTH IMPACTS OF UNIVERSAL CREDIT: PROTOCOL FOR A MIXED METHODS STUDY

Peter Craig, MRC/CSO Social and Public Health Sciences Unit, University of Glasgow, Berkeley Square, 99 Berkeley Street, Glasgow G3 7HR, UK, peter.craig@glasgow.ac.uk*

Ben Barr, Department of Public Health, Policy and Systems, University of Liverpool, UK

Andrew Baxter, MRC/CSO Social and Public Health Sciences Unit, University of Glasgow, UK

Heather Brown, Finnish Institute of Health and Welfare, Finland

Mandy Cheetham, Department of Nursing, Midwifery and Health, Northumbria University, UK

Marcia Gibson, MRC/CSO Social and Public Health Sciences Unit, University of Glasgow, UK

Srinivasa Vittal Katikireddi, MRC/CSO Social and Public Health Sciences Unit, University of Glasgow,UK

Suzanne Moffatt, Population Health Sciences Institute, Newcastle University, UK

Stephanie Morris, Population Health Sciences Institute, Newcastle University, UK

Luke Munford, Health Organisation, Policy and Economics Research Team, University of Manchester, UK

Matteo Richiardi, Centre for Microsimulation and Policy Analysis, University of Essex, UK

Matt Sutton, Health Organisation, Policy and Economics Research Team University of Manchester, UK

David Taylor Robinson, Department of Public Health, Policy and Systems, University of Liverpool, UK

Sophie Wickham, Department of Public Health, Policy and Systems, University of Liverpool, UK

Huasheng Xiang, Population Health Sciences Institute, Newcastle University, UK

Clare Bambra, Population Health Sciences Institute, Newcastle University, UK

*Corresponding author

Word count: 4490

For peer review only

ABSTRACT

Introduction

The UK social security system is being transformed by the implementation of Universal Credit, which combines six existing benefits and tax credits into a single payment for low-income households. Despite extensive reports of hardship associated with the introduction of Universal Credit, no previous studies have comprehensively evaluated its impact on mental health. Because payments are targeted at low-income households, impacts on mental health will have important consequences for health inequalities.

Methods and analysis

We will conduct a mixed methods study. Work package 1 will compare health outcomes for new recipients of UC with outcomes for legacy benefit recipients in two large population surveys, using the phased rollout of UC as a natural experiment. We will also analyse the relationship between the proportion of UC claimants in small areas and a composite measure of mental health. WP2 will use data collected by Citizen's Advice to explore the socio-demographic and health characteristics of people who seek advice when claiming UC and identify features of the claim process that prompt advice-seeking. WP3 will conduct longitudinal in-depth interviews with up to 80 UC claimants in England and Scotland to explore reasons for claiming and experiences of the claim process. Up to 30 staff supporting claimants will also be interviewed. WP4 will use a dynamic microsimulation model to simulate the longer-term health impacts of different implementation scenarios. WP5 will undertake cost-consequence analysis of the potential costs and outcomes of introducing UC and cost-benefit analyses of mitigating actions.

Ethics and dissemination

We obtained ethical approval for the primary data gathering from the University of Glasgow, College of Social Sciences Research Ethics Committee, application number 400200244. We will use our networks to actively disseminate findings to UC claimants, the public, practitioners and policy-makers, using a range of methods and formats.

Registration details

The study is registered with the Research Registry: researchregistry6697.

Keywords

Universal Credit, mental health, mixed methods

For peer review only

STRENGTHS AND LIMITATIONS OF THIS STUDY

- Implementation of Universal Credit is in progress, ruling out a randomised trial, so we exploit the large scale natural experiment generated by phased implementation of the new benefit.
- We use large-scale population surveys to estimate impacts, but the fixed sample sizes limit the range of subgroup analyses we can undertake.
- Our mixed methods design enables us to use information about the experiences of people claiming benefit and seeking advice to inform the detailed design of the quantitative analyses.
- We consulted extensively with UC recipients and organisations representing and providing support for UC recipients in designing the study to ensure that we address questions that matter to UC recipients and organisations that support them.

For peer review only

INTRODUCTION

Social security policies account for up to one quarter of GDP and 40% of public expenditure in high income countries[1] and are a key determinant of income and health inequalities.[2] In the UK, Universal Credit (UC) is transforming social security for working age people by combining six existing benefits and tax credits (known as legacy benefits) into a single monthly payment. UC is designed to improve work incentives for people on low incomes but has been criticised for causing hardship due to the job-seeking requirements and other conditions related to eligibility, and to the way that claims are managed and payments made. UC is being introduced gradually, with rollout due to be complete in 2024.[3] Research to date has not provided a comprehensive picture of UC's impact on population health or health inequalities. Our proposed study will be the first to do so, by examining the impact of the introduction of UC on mental health and wellbeing, and how these effects are moderated by variation in implementation across England, Wales and Scotland.

In preparing our proposal we updated and extended the literature searches from a recently published study of the impact of UC on psychological distress[4] by members of our team, to include papers published in 2019-20. We searched six bibliographic databases (MEDLINE (number of hits=0), PubMed (5), Scopus (5), PsycInfo (0), Social Science Citation Index (8), EconLit (3), using the terms ("universal credit" AND "mental health" OR "wellbeing" OR "well-being" OR depress* OR anxiety OR "psychiatric disorder*" OR "common mental disorder*" OR "psych* morbidity"). We also searched two preprint servers (Socarxiv (2), Medarxiv (1). We found 17 papers, but no new studies, other than our own,[2] that dealt with the health impacts of UC.

Several reports from public bodies draw attention to possible health harms. Based on evidence from local authorities and charities, the UK Parliament's Public Accounts Committee has raised concerns about financial hardship associated with the five week wait for the first payment, compounded by delays in processing new claims.[5] The UN Special Rapporteur on extreme poverty and human rights highlighted difficulties faced by 'poorer and more vulnerable households' in negotiating the online application process, concluding that 'UC was harming many claimants' mental health, finances, and work prospects.'[6] Doctors' organisations have reported increased consultations in General Practices following the transition to UC.[7] Significant concerns have been raised about the impact of UC on rising child poverty in the UK.[8] Qualitative studies have reported 'widespread and deeply negative impacts on wellbeing.'[9] In our own research,[10, 11] claimants reported that the threat of sanctions, delays in payment and financial insecurity associated with UC harmed their physical and mental health. There has been one quantitative study of health impacts to date, again by members of our team.[4] This study compared changes in mental health among unemployed

For peer review only

people in areas where UC had been implemented with areas where legacy benefits remained in place, finding a seven-percentage point increase in psychological distress in the former.

Although existing evidence suggests mainly adverse effects, UC may improve health if it provides a higher or more dependable income for recipients than existing benefits, or if it supports paid work. Research conducted by the Department for Work and Pensions' (DWP) found that more intensive support for job-seeking under UC increased earnings[12] and helped unemployed claimants return to work sooner.[13]

To date, the evidence of health impacts relates to a period when UC had only been implemented for some groups of new claimants. The overall health and equity impacts may change markedly as legacy benefit recipients migrate to UC. Substantial but temporary changes in the way UC is administered were made in response to the economic shock associated with the COVID-19 lockdown measures in early 2020. Conditionality and some forms of debt recovery were suspended for three months and have since remained well below pre-pandemic levels. The standard cash allowance (the most a household could receive, before taking income and savings into account) was raised by £1000 per annum from April 2020 until October 2021[14] A very large number of new claims were made and processed in the early months of the lockdown. Claims rose 9-fold, to 500,000 per week in early April 2020, and the caseload doubled from 3 to 6 million, before new claims returned to previous levels by July 2020.[15] A new cohort of UC recipients has been created whose composition is likely to differ significantly from the pre COVID-19 caseload (for example, they are less likely to have received benefits in the past and more likely to have savings).[16] As well as estimating the impact of UC on population mental health, we shall explore the experiences of these new recipients to gain insights into whether differences in health impacts might be expected.

Our objectives are: (1) to measure the impacts on mental health and wellbeing among adults and children affected by UC, compared to a legacy-benefits comparator group; to explore how impacts are distributed and effects vary between England/Wales and Scotland, and the implications of the COVID-19 related changes to UC for the health of recipients; (2) to identify features of the UC claim and payments process associated with health difficulties for claimants, and to explore inequalities within and between regions in the pattern of advice-seeking from Citizens Advice; (3) to explore the ways in which the experience of claiming and managing UC affects claimants' health, and how experiences differ between England/Wales and Scotland; (4) to develop a microsimulation model to investigate possible income, employment and health consequences of differing UC implementation scenarios, up to ten years post-implementation; (5) to measure the cost-consequence of UC, analyse

For peer review only - http://bmjopen.bmj.com/site/about/guidelines.xhtml

its distributional consequences and conduct cost-benefit analyses of moderating/mitigating measures implemented in Scotland or by specific Local Authorities.

METHODS AND ANALYSIS

Study design

We will undertake a mixed methods study with five closely linked work packages. Work package (WP) 1 will take a natural experimental approach to identify the effects of UC on mental health and wellbeing using two large-scale population surveys, the UK Household Longitudinal Study (UKHLS)[17] and the Annual Population Survey (APS).[18] We shall use the information about the phased implementation of UC that is made publicly available via the DWP's Stat-Xplore system[19] to define exposed and unexposed groups and compare changes in the health of survey respondents exposed to UC, with changes in the health of respondents living in areas where legacy benefits still apply. We will also use a longitudinal neighbourhood dataset for 42,000 small areas (Lower Layer Super Output areas in England and Wales; Datazones in Scotland), the NIHR North West Coast Applied Research Collaboration – Place-based Longitudinal Data Resource.[20] The dataset includes monthly measures indicating which population groups (e.g., new claimants, existing claimants, people with/ without children, people with disabilities), were affected by the phased rollout of UC in each neighbourhood in each time period. To identify any area-level mental health effects of UC, we will utilise a composite neighbourhood mental health measure – the Small Area Mental Health Index (SAMHI), available annually for the same small areas, but adapted to include Scotland.[21] Using the COVID-19 waves of the UKHLS, we shall compare mental health among new UC claimants, other respondents who have experienced income losses and existing UC claimants. These analyses will be primarily descriptive rather than designed to formally identify impacts.

WP2 will analyse administrative data collected by Citizens Advice (CA) on the characteristics of people seeking help in connection with UC claims between 2017-2020, a survey conducted in the North East of England on financial stress associated with the UC application process in September/October 2019, and a linked dataset derived from the Liverpool 'advice on prescription' service, which combines CA data on advice-seeking with information about use of health and social care services.

WP3 comprises two qualitative studies: (i) a longitudinal qualitative and photovoice[22] study of UC claimants and (ii) qualitative interviews with DWP JobCentre Plus staff who support claimants through the process of claiming and front line staff from education, local government, housing and the voluntary and community sectors who support people claiming UC. We will seek to interview

For peer review only

claimants at two timepoints, 18-24 months apart, to explore how the experience of claiming and receiving UC is influenced by other changes in life circumstances, such as transitions into or out of work, changes in relationship status, birth of children or changes in housing arrangements. Use of photovoice methods, alongside more conventional qualitative methods, will enable the follow-up interviews to be participant-led.

WP4 will develop a stochastic dynamic microsimulation model of the longer-term health impacts of UC, building on an existing static microsimulation model of income and welfare policy (UKMOD, www.microsimulation.ac.uk/ukmod), and incorporating information about effects and mediators from the other project work packages. We shall use the model to simulate the possible health impacts of different UC implementation scenarios, and to explore how these differ between subgroups of the working age population, defined by personal characteristics, household type or geography.

WP5 will undertake a cost-consequence analysis of Universal Credit (UC) and assess its distributional impacts by health, income, and other individual and area-level characteristics. We will identify and estimate the magnitude of the wide range of potential positive and negative consequences of introducing UC. We will provide, where appropriate, monetarised valuations of these effects and detail which sectors and government departments experience them. Costs and consequences associated with implementing UC will be compared to a baseline scenario of continued legacy benefits. We will also undertake cost-benefit analyses of a range of actions that have been implemented in Scotland or locally within England to mitigate possible negative impacts of implementing UC.

Intervention

UC combines six existing benefits and tax credits for people who are unemployed or on a low income into a single monthly payment. It is intended to encourage an early return to work by incorporating support for job-seeking and removing disincentives associated with high marginal withdrawal rates under the legacy benefits. The differences between UC and the legacy benefits are summarised in figure 1.

Figure 1 here

[UC began to be used for new claims in October 2013, with rollout for new unemployed claimants complete by December 2018. Existing claims for legacy benefits and tax credits will be transferred to UC in a process of 'managed migration'. The process began with a geographically limited pilot which

For peer review only

was suspended early in 2020, but is expected to recommence in 2022, although how migration will now be managed is not yet known.

UC may improve recipients' health via increased income and employment, but some features of its design and delivery may be harmful. The application of conditionality and sanctions, the minimum five week wait for the first payment, which may in practice be much longer,[3] the two child limit and benefit cap, and the inclusion of support for housing costs within a single monthly household-level payment made to one individual, have all attracted concern. Some households, such as working families living in rented accommodation, will have higher incomes from UC than they would receive from legacy benefits, but others, including people receiving disability benefits, self-employed people with low earnings, and some working lone parents who were formerly receiving tax credits, may lose income.[23] UC is a UK-wide system but in Scotland, under arrangements known as 'Scottish Choices,' claimants can opt for two-weekly payments, or to have the housing element paid direct to their landlord.[24, 25]

The effects on health of the transition to UC are therefore likely be complex and geographically variable, with some households benefitting from improved living standards and greater financial security, but others facing an increase in hardship, insecurity and debt.[26] Effects may be direct, for example by increasing stress, or be mediated by changes in behaviours such as smoking, drinking, or criminal activity.[27] Children's health likewise will be affected directly by changes in household living standards,[28] for example in relation to diet or warmth, but also by effects operating through parental stress and consequent changes in parenting and other behaviours.[29]

As noted above, substantial but temporary changes in the way UC is administered have been made in response to the economic shock associated with the COVID-19 lockdown measures. As well as estimating the overall impact on UC on population mental health, we shall explore the experiences of these new UC recipients to gain insights into whether differences in health impacts might be expected.

Sample selection

In WP1 we will analyse data on people of working age and their children from 2009-2022 in the UKHLS, and on people of working age from 2011-2024 in the APS. The UKHLS and APS use stratified clustered random probability samples of the UK population. The CA surveys (WP2) include adults in England who have contacted CA for assistance with claiming UC from 2017 onwards. For a sub-sample of respondents from North East England a survey was conducted by CA was in August 2019 and a follow up questionnaire was administered in October 2019 to all individuals who had made a UC claim to understand the characteristics of people who experience financial hardship. The 'advice

For peer review only

on prescription service' data is available for people engaging with CA Liverpool from 2014, but linked data will span 2010 to 2023.

The samples of UC claimants for WP3 will be drawn from two large urban areas, Tyne and Wear (population 1.1 million) in North East England and the Glasgow City Region (population 1.2 million) in Scotland. A number of recruitment strategies will be used, including via gatekeepers[8, 9] in public, voluntary and community sector organisations, workplaces and trades unions, and via social media and leaflets placed in community locations such as libraries. We will aim for a maximum variation sample, taking account of age, gender, ethnicity, household composition, education level, health, disability, employment and housing status. The sample will include people who have been on UC for some time, new claimants and those transferring to UC from legacy benefits. We will include people who were claiming UC prior to March 2020, people who claimed UC during the UK's lockdown period and those who have become claimants later due to the economic impact of the pandemic. We will recruit staff from a range of organisations in each locality via our extensive professional networks in each conurbation.

Sample size and statistical power

The UKHLS includes approximately 40,000 households who are surveyed annually. The 2009-2022 survey datasets will include approximately 280,000 observations on around 52,000 adults and 30,000 observations on 5,000 children. To identify the impact of UC, we will conduct difference-in-difference (DiD) analyses, comparing members of households newly exposed to UC as rollout proceeds, with households who are unlikely to be exposed. Focusing on child health outcomes, because the numbers available are smaller, in the UKHLS datasets from 2009-18 we will have 300 children in the intervention group and 3300 in the control group, providing 80% power to detect an 8% relative increase in SDQ scores. This is a markedly smaller effect than the 20% relative increase in the prevalence of psychological distress we found in our previous study[4] of the impact of Universal Credit on adult mental health up until 2018, so provides a conservative estimate of the power of the proposed study. The sample size in the UKHLS COVID-19 surveys is approximately 10,000, of whom approximately 4,000 reported substantial (>5%) income losses, and around 300 reported new UC claims in the April survey wave. Currently, nine waves of data are available, from April 2020 to September 2021, with questions about UC included in waves 1, 2, 4, 6, 8 and 9.[30]

The APS collects self-assessed health and wellbeing data on approximately 180,000 working age people per year, so provides good power for England-Scotland comparisons and other subgroup analyses. Our analysis of area level mental health effects will utilise annual data from a longitudinal

For peer review only

dataset of 42,000 neighbourhoods from 2011 to 2022, or approximately 504,000 neighbourhood-years.

The CA survey dataset contains information on approximately 280,000 people who seek advice. The longitudinal survey contains information on 250 people, with a response rate of 85%. The advice on prescription dataset contains information on approximately 5000 people each year since 2014.

For the qualitative study, we will recruit up to 80 UC claimants aged 18–66 (state pension age), divided equally between the two study sites. We will seek to re-interview 40 first round respondents 18-24 months after their first interview, a sub-sample of interviewees using photovoice methods.[22] We will aim to interview up to 15 staff from a range of organisations in each study site.

WP4 and 5 draw on data from WP1-3 above and from the additional sources listed in the analysis plan below.

Outcomes

Our primary focus is on mental health and wellbeing. Mental health is an important dimension of overall health and wellbeing, that is sensitive to the effects of changes in employment or income,[31, 32] and an important determinant of physical health. Parental mental health is also an important mediator of the impact of poverty on child health.[33] In the survey analyses (WP1) we shall use standard measures of mental health and wellbeing, such as the Mental Component Summary (MCS) of the 12-item Short-Form Health Survey (SF-12) for adults, the Strengths and Difficulties Questionnaire (SDQ) for children,[34] and the Office for National Statistics (ONS) ONS4 measure of wellbeing (life satisfaction, sense that life is worthwhile, happiness and anxiety).[35] Secondary outcomes include other mental and physical health measures available in the surveys. In the neighbourhood-level analyses we will use a composite mental health measure, the Small Area Mental Health Index (SAMHI).[21] In the CA surveys in WP2 we shall use presence of a self-reported long-term mental health condition. WP4 and WP5 will use similar outcomes to WP1, and WP5 will also explore the costs and non-health consequences of implementing UC. In WP3 we shall explore other health outcomes identified as important by respondents.

Analysis plan

To identify the effects of UC in the UKHLS and APS datasets (WP1) we will fit DiD models, with terms for group and period to define respondents who are exposed or unexposed to UC, depending on their socio-demographic characteristics, location and date of interview, and a group by period interaction term to identify the effect of UC. We will add individual and area-level covariates to account for time-varying confounders[36] and fit mixed-models to account for clustering of

For peer review only

observations within households and areas. Sensitivity analyses will be conducted using alternative definitions of exposed and unexposed populations. We will test the validity of the key identifying assumptions, such as common pre-intervention trends, and use additional (e.g., matching) methods if needed. We will also conduct falsification tests using placebo outcomes to test for specificity of effects.

For the SAMHI analyses we will use fixed-effect panel regression methods to investigate whether increases in UC in each LSOA are associated with changes in mental health problems, while controlling for time-varying confounders (e.g. local government expenditure)[37].We will check the robustness of the results by using alternative ways of estimating effects, such as continuous DiD and synthetic control methods[38] to compare outcomes in areas where UC has been introduced and areas not yet exposed.

To investigate the extent to which changes in income, employment status, and financial housing difficulties (such as rent arears) mediate the effects of the introduction of UC on health outcomes, we will undertake causal mediation analysis adjusting for time-varying confounding.[39, 40, 41] Subgroup analysis will investigate differences in health impacts between Scotland and the rest of GB, or between areas with differing labour market characteristics. By stratifying the analysis before, during and after the pandemic we will investigate whether moving onto UC had the same effect during these different time periods, when the policy and wider social context were very different. Using the COVID-19 waves of the UKHLS, we shall compare mental wellbeing among new UC claimants, other respondents who have experienced income losses and existing UC claimants. These analyses will be primarily descriptive rather than designed to formally identify impacts.

In WP2 we will carry out descriptive analyses of the England-wide CA survey data to summarise the health and socio-demographic characteristics of people seeking advice in applying for UC, and explore differences at the local authority level in key demographic and health characteristics of advice seekers. We will use the longitudinal CA dataset to describe the socio-demographic and health characteristics of people who are most and least likely to experience financial insecurity as a result of the five week wait for the first payment. We will also apply longitudinal multivariable regression to the 'advice on prescription' linked dataset, similar to the methods utilised in WP1. We will use the cross-sectional national data from March 2020 onwards to explore changes in the socio-demographic and health profile of people seeking advice with UC claims following the COVID-19 pandemic.

In WP3, we will apply thematic content analysis to the longitudinal qualitative data,[42] using NVivo software to assist data management, retrieval and sharing between members of the research team.

For peer review only

A comparative analysis across time, locality and participant characteristics will be undertaken to identify possible pathways linking the experience of claiming UC with health outcomes, and how they vary between claimants and between study sites. This will enable identification of differences of experience by locality and in-depth analysis of characteristics including gender, age, and employment status, as well as facilitating comparisons of the experiences of longer term (pre-COVID) and post-COVID claimants. Photovoice data will be coded and incorporated within the analysis of the interview material.[22] Interviews with staff will also be analysed thematically.

In WP4 we will first create a dynamic, synthetic population that reflects the GB population, using data from UKHLS, APS, census data, population projections, mortality estimates and the Family Resources Survey. We will develop a new stochastic dynamic microsimulation model based on the existing UKMOD income and welfare policy model,[43] coupled with estimates of the relationship between UC, income, employment and health. We will use the model to simulate the effects on health of different UC implementation scenarios compared with three baseline scenarios: the continuation of legacy benefits; UC as it existed in the early months of the COVID-19 pandemic; and UC in its contemporary form (i.e., at the time of running the model). Implementation scenarios will include varying levels of standard allowance, conditionality, payment frequency, etc. Under each scenario, we will transition the synthetic population forward in one-year increments for up to ten years, so that we can compare the effects on health and health inequalities of the different policy choices. We will check the robustness of the model by comparing simulated health outcomes with known outcomes from UKHLS waves that were not used to parameterise the model and from other surveys such as the Scottish Health Survey. To allow for the uncertainty in the sample-based parameter estimates used to build the model, we will use Monte Carlo simulation (10,000 runs) to derive 95% credible intervals.

WP5 comprises three linked analyses: ex post cost-consequence and distributional cost-consequence analyses of the implementation of UC, and an ex ante cost benefit analysis of measures to mitigate against possible adverse effects of UC. We will follow HM Treasury Green Book[44] guidelines for economic evaluation, to enable comparability with other evaluations of UK Government policies. We will obtain measures of costs of implementing legacy benefits, UC and mitigating measures through liaison with project partners. We will consider a wide range of consequences, informed by liaison with stakeholders and the findings of WPs 1-3. They will include, but not be limited to, health and wellbeing; income (at both individual and household level); employment; economic productivity (Gross Value Added); and use of health and care services. We will use estimates of the short term effects of UC from WP1, supplemented by additional analyses using similar methods as far as possible, but considering alternatives (e.g. lagged dependent

For peer review only

variables, synthetic controls) where DiD assumptions are not met.[45, 46] Economic, health and wellbeing outcomes will be monetarised where appropriate using standard methods[47, 48] The initial time horizon for the accrual of costs and consequences will be ten years (consistent with WP4), though we will consider alternative time-horizons informed by discussions with stakeholders.

The overall duration of the study is 52 months from May 2021-August 2025. Primary data gathering began in January 2022 and will be completed in September 2024.

Public Involvement and Engagement

In designing the study we consulted widely with organisations representing and providing support for UC claimants. Strong public involvement and engagement (PIE) will be maintained throughout the study using a range of methods and with specialist support from the North East and North Cumbria Applied Research Collaboration. We define 'public' to include current or former UC claimants, users and staff of advice and support services that help UC claimants, and policy makers and practitioners with working knowledge of UC implementation. We shall seek their advice on changes in UC policy and delivery, aspects of research methods, including data gathering, analysis, interpretation and reporting, and their active engagement in the development of payment policies and procedures, fieldwork documents, analysis plans and wider dissemination. Engagement will be co-ordinated by a named PIE lead (MC), and the whole study team will work according to an agreed PIE values framework.

ETHICS AND DISSEMINATION

Ethics

The University of Glasgow is the study sponsor. We have obtained ethical approval for the primary data gathering in WP3 from The University of Glasgow College of Social Sciences Research Ethics Committee, application number 400200244. We are using a Special Licence version of the APS datasets, under the UK Data Service Secure Lab Project No. 116676. Survey respondents provided informed consent before being interviewed. We shall obtain informed consent from participants before interviewing them in the qualitative study.

Dissemination

As well as presenting findings at academic meetings and publishing findings in peer-reviewed journals we will use our networks to actively disseminate findings to UC claimants, the wider public,

For peer review only

practitioners and policy-makers. Specific impact-focused activities will include: (1) Co-production of non-academic outputs for national and local government actors, provider organisations and advocacy groups e.g. text and video narrative summaries; 'policy briefings'; infographics; research digests; (2) Photo/text exhibition for a national public engagement tour of libraries, community centres, museums, galleries; (3) Targeted, interactive work-in-progress and policy briefing sessions for varied policy and practice audiences; (4) Stakeholder workshops and learning events for key interest groups; (4) Joint research meetings involving contributions from academics, policy and practice and public; (5) A purpose-built website to disseminate results via policy briefs, blogs and infographics (6) 'open source' versions of the WP4 policy models to enable government policy analysts to use them to inform future decision-making and for other researchers to apply our methodology into other areas; (7) Public engagement sessions via science festivals and various university initiatives e.g. Pint of Science, Café Scientifique; (8) Social media and traditional media press releases of key results.

For peer review only

Contributorship statement

PC, BB, HB, MC, MG, SVK, SMoffatt, LM, MR, MS, DTR, SW and CB contributed to the design and planning of the study and to acquisition of funding. PC wrote the first draft of the paper. PC, BB, AB, HB, MC, MG, SVK, SMoffatt, SMorris, LM, MR, MS, DTR, SW, HX and CB contributed to revising the manuscript and approving the final version for publication, and agree to be accountable for all aspects of the work.

Competing interests

The authors declare no competing interests.

Funding

This research is supported by the UK National Institute of Health Research Public Health Research Programme, grant number NIHR131709. PC, MG and SVK acknowledge funding from the UK Medical Research Council (MC_UU_00022/2) and Scottish Government Chief Scientist Office (SPHSU17). SVK acknowledges funding from a NRS Senior Clinical Fellowship (SCAF/15/02) and the European Research Council (949582). CB and MC acknowledge funding from ARC North East North Cumbria (NIHR200173), and CB from the National Prevention Research Initiative for the Systems science in Public Health and Health Economics Research consortium (MR/S037578/1). LM and MS acknowledge funding from ARC Greater Manchester (NIHR200174). SW acknowledges funding from a Wellcome Trust Society and Ethics Research Fellowship (200335/Z/15/Z). BB and DT-R are funded by the National Institute for Health Research (NIHR) School for Public Health Research (SPHR), Grant Reference Number PD-SPH-2015. DT-R is also funded by the Medical Research Council (MRC) on a

Clinician Scientist Fellowship (MR/P008577/1). BB is also supported by the National Institute for Health Research Applied Research Collaboration North West Coast (ARC NWC).

For peer review only

REFERENCES

[1] Eurostat. Government expenditure on social protection. https://ec.europa.eu/eurostat/statistics-explained/index.php?title=Government_expenditure_on_social_protection (last accessed 6 January 2022).

[2] Mackenbach JP, Bopp M, Deboosere P, et al. Determinants of the magnitude of socioeconomic inequalities in mortality: A study of 17 European countries. Health Place 2017;4744-53.

[3] National Audit Office. Rolling out Universal Credit. 2018. https://www.nao.org.uk/wp-content/uploads/2018/06/Rolling-out-Universal-Credit.pdf (last accessed 6 January 2022).

[4] Wickham S, Bentley L, Rose T, et al. Effects on mental health of a UK welfare reform, Universal Credit: a longitudinal controlled study. Lancet Public Health, 2020;5:e157-e164.

[5] House of Commons Committee of Public Accounts. Universal Credit. Sixty-Fourth Report of Session 2017-19. HC 1183, 26 October 2018.

[6] Statement on Visit to the United Kingdom, by Professor Philip Alston, United Nations Special Rapporteur on extreme poverty and human rights. London, 16 November 2018.

[7] Arie S, Doctors' concerns over universal credit are mounting. BMJ 2018;363.

[8] Child Poverty Action Group. Universal Credit: What needs to change to reduce child poverty and make it fit for families? CPAG, June 2019.
https://cpag.org.uk/sites/default/files/files/policypost/Universal%20credit%20-%20what%20needs%20to%20change_0.pdf (last accessed 20/01/2022).

[9] Wright S, Dwyer P, Jones K, et al. Final findings: universal credit. Welfare Conditionality, Sanctions, Support and Behaviour Change. 2018. http://www.welfareconditionality.ac.uk/wp-content/uploads/2018/05/40414-Universal-Credit-web.pdf

[10] Cheetham M, Moffatt S, Addison M, et al Impact of Universal Credit in North East England: a qualitative study of claimants and support staff. BMJ Open 2019;9:e029611.

[11] Cheetham M, Gorman S, Gibson E, et al. "It's not about telling people to eat better, stop smoking or get on the treadmill." In: Newbury-Birch D and Allan K, eds. *Co-creating and co-producing Research Evidence. A Guide for practitioners and academics in health, social care and education settings.* Abingdon: Routledge, 2020.

[12] Department for Work and Pensions. Universal Credit: in work progression randomised controlled trial. https://www.gov.uk/government/publications/universal-credit-in-work-progression-randomised-controlled-trial (last accessed 20/01/2022).

[13] Department for Work and Pensions. Estimating the Early Labour Market Impacts of Universal Credit. DWP Ad Hoc Research Report No. 19. February 2015.

For peer review only

[14] House of Commons Library, Coronavirus. Support for Household Finances. 22 June 2020. https://commonslibrary.parliament.uk/research-briefings/cbp-8894/ (last accessed 20/01/2022).[15] Department for Work and Pensions. Universal Credit Statistics: 29 April 2013 to 9 July 2020. https://www.gov.uk/government/statistics/universal-credit-statistics-29-april-2013-to-9-july-2020/ (last accessed 20/01/2022).

[16] House of Lords Economic Affairs Committee. Universal Credit isn't working: proposals for reform 2nd Report of Session 2019-21. HL Paper 105, 31 July 2020 https://publications.parliament.uk/pa/ld5801/ldselect/ldeconaf/105/10502.htm (last accessed 20/01/2022).

[17] Understanding Society. COVID-19. https://www.understandingsociety.ac.uk/topic/covid-19 (last accessed 20/01/2022).

[18] Office for National Statistics. Annual Population Survey (APS) QMI. https://www.ons.gov.uk/employmentandlabourmarket/peopleinwork/employmentandemployeetypes/methodologies/annualpopulationsurveyapsqmi (last accessed 20/01/2022).

[19] Department for Work and Pensions. Stat-Xplore. 2019. https://stat-xplore.dwp.gov.uk/webapi/jsf/login.xhtml (last accessed 20/01/2022).

20 Place-based Longitudinal Data Resource. https://pldr.org/ (last accessed 06/01/2022).

21 Daras K, Barr B (2021), Small Area Mental Health Index (SAMHI) [Open Dataset], Place-based Longitudinal Data Resource, DOI: 10.17638/datacat.liverpool.ac.uk/1188, last accessed 06/01/2022.

[22] Guell C, Ogilvie D. Picturing community: photovoice and seeking well-being in everyday travel. Qual Res 2015;15:201-218.

[23] Brewer M, Joyce R, Waters T et al. Universal credit and its impact on household incomes: the long and the short of it. https://www.ifs.org.uk/publications/14083 (last accessed 20/01/2022).

[24] Universal Credit (Scottish choices). https://www.gov.scot/policies/social-security/universal-credit/ (last accessed 20/01/2022).

[25] Child Poverty Action Group (2019) Universal Credit Scottish Choices. 18/2/19 https://cpag.org.uk/scotland/welfare-rights/scottish-benefits/universal-credit-scottish-choices (last accessed 16.07.20).

[26] Bennett F, Sung S. Money matters: using qualitative research for policy influencing on gender and welfare reform, Innovation: Eur J Soc Sci Res 2014;27:5-19.

[27] d'Este R, Harvey A. Universal Credit and Crime. SSRN, October 14, 2020. https://ssrn.com/abstract=3642502 or http://dx.doi.org/10.2139/ssrn.3642502 (last accessed 20/01/2022).

For peer review only

[28] Stewart K, Reeves A and Patrick R. A time of need: Exploring the changing poverty risk facing larger families in the UK. CASE Working Paper 224, July 2021. https://sticerd.lse.ac.uk/dps/case/cp/casepaper224.pdf (last accessed 20/01/2022).

[29] Pearce A, Dundas R, Whitehead M, et al. Pathways to inequalities in child health. Arch Dis Child 2019;104:998-1003.

[30] Understanding Society. COVID-19. https://www.understandingsociety.ac.uk/documentation/covid-19 (last accessed 10/01/2022)

[31] Reeves A, Fransham M, Stewart K et al.Did the introduction of the benefit cap in Britain harm mental health? A natural experiment approach. CASE Working Paper 221, November 2020. https://sticerd.lse.ac.uk/dps/case/cp/casepaper221.pdf (last accessed 20/01/2022).

[32] Reeves A, McKee M, Mackenbach J et al. Introduction of a national minimum wage reduces depressive symptoms in low wage workers; a quasi-natural experiment in the UK. Health Econ 2016;26:639–655 (last accessed 20/01/2022).

[33] Wickham S, Whitehead M, Barr B, et al. The effect of a transition into poverty on child and maternal mental health: a longitudinal analysis of the UK Millennium Cohort Study. Lancet Public Health 2020; 5: e157–64 [34] Goodman, R. (1997), The Strengths and Difficulties Questionnaire: A Research Note. J Child Psychol Psychiatr 1997;38:581-586.[35] ONS. Personal well-being user guidance. https://www.ons.gov.uk/peoplepopulationandcommunity/wellbeing/methodologies/personalwellbeingsurveyuserguide (last accessed 20/01/2022).

[36] Daniel RM, Cousens SN, De Stavola BL, et al. Methods for dealing with time-dependent confounding. Stat Med 2013;32:1584–618.

[37] Barr B, Taylor-Robinson D, Stuckler D, et al. 'First, do no harm': are disability assessments associated with adverse trends in mental health? A longitudinal ecological study. J Epidemiol Community Health 2016;70:339-345

[38] Robbins MW, Saunders J, Kilmer B. A Framework for Synthetic Control Methods With High-Dimensional, Micro-Level Data: Evaluating a Neighborhood-Specific Crime Intervention. J Am Stat Assoc 2017;112:109–26.

[39] VanderWeele TJ. Explanation in Causal Inference: Methods for Mediation and Interaction. New York: Oxford University Press, 2015.

[40] Imbens GW, Rubin DB. Causal Inference for Statistics, Social and Biomedical Sciences: an Introduction. New York: Cambridge University Press, 2015.

[41] Hernán MA, Robins JM. Estimating causal effects from epidemiological data. Journal of epidemiology and community health 2006;60:578–86.

For peer review only

[42] Neale B What is longitudinal qualitative research ? London: Bloomsbury Academic, 2018
[43] Richiardi M, Collado D and Popova D. UKMOD –A new tax-benefit model for the four nations of the UK. Int J Microsimul, forthcoming. 2021
[44] HM Treasury. The Green book. Central Government Guidance on Appraisal and Evaluation. HM Treasury, 2018. https://assets.publishing.service.gov.uk/government/uploads/system/uploads/attachment_data/file/685903/The_Green_Book.pdf (last accessed 20/01/2022).
[45] O'Neill S, Kreif, N, Grieve, R et al. Estimating causal effects: considering three alternatives to difference-in-differences estimation. Health Serv Outcomes Res Method 2016;16:1–21.
[46] O'Neill S, Kreif N, Sutton M, et al. A comparison of methods for health policy evaluation with controlled pre-post designs. Health Serv Res. 2020;55:328-338.
[47] NICE. How NICE Measures Value for Money in Relation to Public Health Interventions. 2013. https://www.nice.org.uk/Media/Default/guidance/LGB10-Briefing-20150126.pdf (last accessed 20/01/2022).
[48] Shah K, Praet C, Devlin N, et al. Is the Aim of the English Health Care System to Maximize QALYs? J Health Serv Res Policy 2012;17(3): 157–63.

For peer review only

Figure Legends

Figure 1 Universal Credit and legacy benefits (Source: Reference [2])

For peer review only

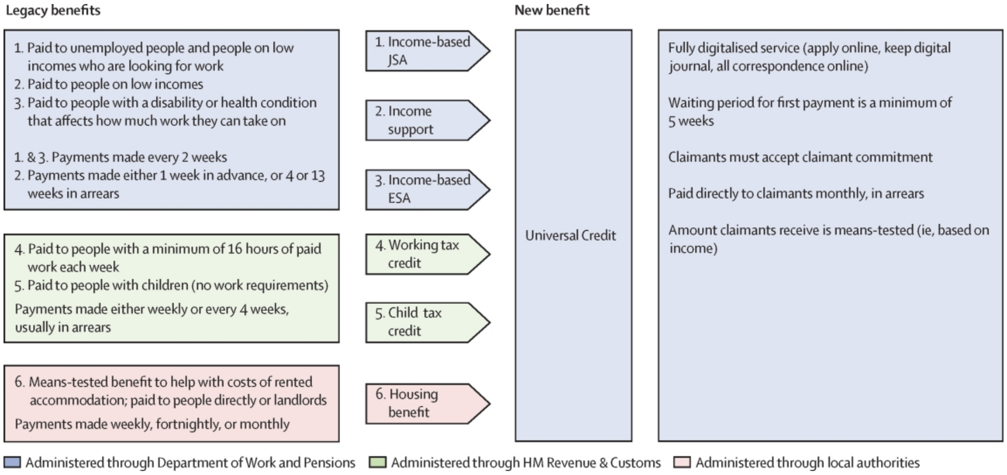

Figure 1 Universal Credit and legacy benefits (Source: Reference [2])

211x98mm (500 x 500 DPI)
