# Supplementary material for: Evaluation of the mental health impacts of Universal Credit: protocol for a mixed methods study
Source: BMJ Open. 2022 Apr 8;12(4):e061340. doi: 10.1136/bmjopen-2022-061340 (PMC8996017; doi:10.1136/bmjopen-2022-061340)
Supplement: Reviewer comments [file bmjopen-2022-061340.reviewer_comments.pdf]

PEER REVIEW HISTORY

BMJ Open publishes all reviews undertaken for accepted manuscripts. Reviewers are asked to complete a checklist review form (**http://bmjopen.bmj.com/site/about/resources/checklist.pdf**) and are provided with free text boxes to elaborate on their assessment. These free text comments are reproduced below.

ARTICLE DETAILS

**TITLE (PROVISIONAL)**	EVALUATION OF THE MENTAL HEALTH IMPACTS OF UNIVERSAL CREDIT: PROTOCOL FOR A MIXED METHODS STUDY
**AUTHORS**	Craig , Peter; Barr, Benjamin; Baxter, Andy; Brown, Heather; Cheetham, Mandy; Gibson, Marcia; Katikireddi, Srinivasa; Moffatt, Suzanne; Morris, Steph; Munford, Luke; Richiardi, Matteo; Sutton, Matt; Taylor-Robinson, David; Wickham, Sophie; Xiang, Huasheng; Bambra, Clare

This article was not externally reviewed at BMJ Open. Protocols that have been independently assessed prior to submission to BMJ Open are usually fast-tracked to publication on the grounds that further substantial changes will not be possible. This independent assessment will usually be external, independent review for both a substantial grant award from a non-commercial or government funder and ethics approval.
